# Leonurine Regulates Hippocampal Nerve Regeneration in Rats with Chronic and Unpredictable Mild Stress by Activating SHH/GLI Signaling Pathway and Restoring Gut Microbiota and Microbial Metabolic Homeostasis

**DOI:** 10.1155/2023/1455634

**Published:** 2023-01-07

**Authors:** Pan Meng, Xi Zhang, Dandan Li, Hui Yang, Xiaoyuan Lin, Hongqing Zhao, Ping Li, Yuhong Wang, Xiaoye Wang, Jinwen Ge

**Affiliations:** ^1^Hunan University of Chinese Medicine, Changsha, Hunan, China; ^2^Hunan Key Laboratory of Traditional Chinese Medicine Prevention & Treatment of Depressive Diseases, Changsha, Hunan, China; ^3^The Second People's Hospital of Hunan Province, Changsha, Hunan, China; ^4^The First Hospital of Hunan University of Chinese Medicine, Changsha, Hunan, China; ^5^Hunan Academy of Chinese Medicine, Changsha, Hunan, China

## Abstract

Depression is a highly prevalent and heterogeneous disorder that requires new strategies to overcome depression. In this study, we aimed to investigate whether leonurine modulated hippocampal nerve regeneration in chronic and unpredictable mild stress (CUMS) rats through the SHH/GLI signaling pathway and restoring gut microbiota and microbial metabolic homeostasis. The CUMS rat model was constructed and treated with leonurine. The body weight of rats was recorded, and a series of tests were performed. Western blot was utilized to measure the expression of BDNF and 5-HT in the hippocampus. Then the expression of SHH, GLI, PTCH, and SMO were measured by qRT-PCR and western blot. The colocalization of BrdU+DCX and BrdU+NeuN was evaluated by IF. 16S rDNA high-throughput sequencing was applied to detect the composition and distribution of gut microbiota. The differential metabolites were analyzed by untargeted metabolomics. The correlation between gut microbiota and microbial metabolites was analyzed by Pearson correlation coefficient. After CUMS modeling, the body weight of rats was decreased, and the expression of BDNF and 5-HT were decreased, while the body weight was recovered, and the expression of BDNF and 5-HT were increased after leonurine treatment. Leonurine reversed the reduction in the colocalization of BrdU+DCX and BrdU+NeuN and the reduction in the levels of SHH, GLI, PTCH, and SMO induced by CUMS modeling. Leonurine also restored gut microbiota and microbial metabolites homeostasis in CUMS rats. Furthermore, *Prevotellaceae_Ga6A1_group* was negatively correlated with 3-Oxocholic acid, nutriacholic acid, and cholic acid. Collectively, leonurine regulated hippocampal nerve regeneration in CUMS rats by activating the SHH/GLI signaling pathway and restoring gut microbiota and microbial metabolic homeostasis.

## 1. Introduction

Depression is a highly common and heterogeneous disorder [1]. It is a very common psychiatric disorder, often related to gender, genetic, environmental, and/or psychological causes [2], that affects many people worldwide, hinders every aspect of their lives, and leads to many suicides each year [3]. Stress is a major risk factor for psychiatric disorders, including major depressive disorder, and can induce inflammation, which is known to be dysregulated in depression [4]. Chronic stress induces neuronal death and impairs hippocampal neurogenesis, thus leading to cognitive deficits and depressive-like behaviors [5]. Depression and inflammation promote each other. For some patients with depression, inflammation plays a key role in the pathogenesis of depression; depression also triggers a greater cytokine response to stressors [6]. Therefore, new strategies are needed to overcome depression and inflammation.

Leonurine is an active ingredient of the traditional Chinese medicine (TCM) Herba Leonuri [7]. Leonurine has microcirculation-improving, antioxidant, antiapoptotic, free radical scavenging, and anti-inflammatory effects, and effectively treats cardiovascular disease in various ways. Moreover, the biological activity of leonurine is expected to enable its clinical application [8]. Leonurine could promote neurite outgrowth and neurotrophic activity in nerve cells [9]. Leonurine has been reported to improve cognitive dysfunction by antagonizing excitotoxic glutamate and inhibiting autophagy [10]. Leonurine also reduced depression-like behavior in chronic mild stress mice, which may be mediated at least in part by improving monoamine neurotransmitters and inhibiting neuroinflammation [11]. However, the underlying molecular mechanism of leonurine alleviating depressive symptoms and hippocampal nerve regeneration in chronic and unpredictable mild stress (CUMS) rats is still unclear, and further research is needed.

Sonic hedgehog (SHH) plays an important role in the development of the vertebrate animal central nervous system (CNS), and GLI is its downstream signal molecule [12]. SHH/GLI signaling pathway has been recognized as a key mediator of many fundamental processes in embryonic development, including CNS growth, patterning, and morphogenesis [13]. Previous studies have shown that maternal prenatal chronic unpredictable mild stress led to the upregulation of GSK-3*β*, which in turn inhibited SHH, *β*-catenin, Notch, and brain-derived neurotrophic factor (BDNF) expression, thereby affecting neonatal hippocampal neurodevelopment [14]. Tayyab et al. reported the antidepressant and neuroprotective effects of naringenin via SHH/GLI1 signaling pathway in a CUMS rat model [15]. These studies suggest that SHH/GLI signaling pathway may be closely related to the neural development of CUMS rats, but whether leonurine alleviates depressive symptoms and hippocampal nerve regeneration in CUMS rats through SHH/GLI signaling pathway needs further experiments to determine.

It is well known that antidepressants can modulate the CNS, and the gut microbiota can play a role in depression through the microbiota-gut-brain axis [16]. An imbalance in the microbiota-gut-brain axis reflects the constant bidirectional communication between the CNS and the gastrointestinal tract, which has been used as a hypothesis to explain the pathogenesis of depression [17]. However, the effects of antidepressants on gut microbiota function and composition remain poorly understood. Studies have shown that fecal microbiota transplantation (FMT) affected gut microbiota in CUMS rats and induced higher levels of depressive behavior and neuroinflammation. Gut microbiota exacerbated anxiety and depression-like phenotypes by modulating proinflammatory cytokines in the hippocampus through a dysfunctional microbiota-gut-brain axis [18]. In addition, changes in fecal metabolite and plasma metabolite abundances in CUMS rats were associated with antidepressant behavior and altered hippocampal neurotransmitter levels, and there was a significant correlation between metabolites and gut microbiota [19]. However, whether leonurine attenuates depressive symptoms and hippocampal nerve regeneration in CUMS rats by modulating gut microbiota and microbial metabolic homeostasis needs to be further explored.

Based on the above background, we reasonably and boldly speculate that leonurine may regulate hippocampal nerve regeneration in CUMS rats by activating the SHH/GLI signaling pathway and restoring gut microbiota and microbial metabolic homeostasis. This study enriches the mechanism research of CUMS and provides a new reference for treating CUMS.

## 2. Materials and Methods

### 2.1. Construction of CUMS Rat Model

Male Sprague Dawley (SD) rats of 6-8 weeks old and weighing 250 ± 20 g were purchased from Hunan SJA Laboratory Animal Co., Ltd and raised in the Animal Experiment Center of the Hunan University of Chinese Medicine, maintaining an ambient room temperature of 22 ± 1°C, a humidity of 40-60%, alternates day and night for 12 h/12 h. The rats were fed with standard food and available tap water.

Thirty SD rats were randomly divided into Control, CUMS and Leonurine groups, with 10 rats in each group. CUMS procedure was as follows: Animals were individually housed in acrylic cages for 5 weeks to induce social isolation, while five cycles of stress regimens were administered weekly, including the following stressors: 45° cage tilt (24 h), food deprivation (48 h), cold swimming (5 min at 4°C), tail clipping (1 min, 1 cm from distal end of tail), body restraint (2 h), damp bedding (8 h), and strobe lighting at night [20]. The rats in the CUMS group were administered stressors in random order every day for five consecutive weeks, without the same stressor for two consecutive days. The rats in the Control group were placed in a separate room to avoid contact with the stressed rats, fed normally, and unaffected by any of these stressors throughout the experiment. The rats in the Leonurine group were given 60 mg/kg of leonurine by gavage every day from 7 days before the administration of stressors until the end of the behavior test [21].

Behavioral tests were performed after 4 weeks of CUMS exposure to assess the induction of depression. The body weight of rats was recorded 1 week before modeling and 0, 1, 2, 3, and 4 weeks after modeling. The tumor tissue and stool were obtained and fixed with 4% neutral formaldehyde or stored at -80°C. Rats were sacrificed with 150 mg/kg sodium pentobarbital. This study was approved by the Laboratory Animal Ethics Committee of the Hunan University of Chinese Medicine, and carried out according to the guidelines for the care and use of laboratory animals and the principles of laboratory animal care and protection (LL2020111001).

### 2.2. Sucrose Preference Test (SPT)

SPT was performed according to previous reports in the literature [22, 23]. The SPT included four stages of adaptation, baseline determination, testing and data analysis, which took 8 days. Sugar water preference percentage = sugar water consumption/(sugar water consumption + distilled water consumption).

### 2.3. Open Field Test (OFT)

Animals were placed in a 60 cm × 45 cm × 60 cm box with a bottom consisting of 12 equal 15 cm × 15 cm squares. After the animals were placed in the center grid, the measurement was started, each measurement was 5 min, and each rat only had one behavior measurement. After the measurement was completed, the feces were cleaned, the bottom of the box was wiped with 75% ethanol, and the next measurement was performed. Manual records are used. The measurement indicators were immobility time, the numbers of climbing (two front paws leave the bottom surface), and the number of crossing (the number of 4 paws passing through the grid) within 5 min.

### 2.4. Forced Swimming Test (FST)

In this experiment, rats were placed in an open cylindrical white plastic container (40 cm in diameter, 80 cm in height), 40 cm of water was injected into the container, the temperature was 22-25°C, and the total recording time was 3 min. Immobility time (the time when the rat floated on the water surface with only slight activity, or when the body was perpendicular to the water surface and only the nose was out of the water) was manually recorded.

### 2.5. Hematoxylin-Eosin (HE) Staining

HE staining was performed to detect the morphological changes of brain tissue. The slices were baked at 60°C for 12 h, dewaxed to water, hematoxylin stained for 1-10 min, washed with distilled water, and PBS turned blue. The slices were dyed with eosin for 1-5 min and washed with distilled water. Gradient alcohol (95-100%) was applied for dehydration. After removal, the slices were placed in xylene for 10 min, twice, sealed with neutral gum, and observed under microscope.

### 2.6. Quantitative Real-Time PCR (qRT-PCR)

Briefly, total RNA was extracted by Trizol method, RNA was reverse transcribed into cDNAs using cDNA reverse transcription kit (#CW2569, Beijing ComWin Biotech, China), and the UltraSYBR Mixture (#CW2601, Beijing ComWin Biotech, China) was applied in the ABI 7900 system. Using *β*-actin as an internal reference gene, the relative level of the gene was calculated by the 2^-*ΔΔ*Ct^ method. The primer sequences were as follows: SMO-F: ATGCGTGTTTCTTTGTGGGC, SMO-R: ACACAGGATAGGGTCTCGCT; PTCH-F: CGCCGGGGTTTTTACACTTTC, PTCH-R: GGTCTCTTTGTCTGCCCTGTC; SHH-F: ATCCAAAGCTCGCATCCACT, SHH-R: CGCGTCTCGATCACGTAGAA; GLI-F: GGACTTTCTGGTCTGCCCTTT, GLI-R: GGGTGAGGTACGGATTACGG; *β*-actin-F: ACATCCGTAAAGACCTCTATGCC, *β*-actin-R: TACTCCTGCTTGCTGATCCAC.

### 2.7. Western Blot

Western blot was used to detect the expression of BDNF, 5-hydroxytryptamine (5-HT), SHH, GLI, patched (PTCH), and smoothened (SMO) in the hippocampus. Total protein was extracted from tissues using RIPA lysis buffer. BCA protein assay kits were used for protein quantification. SDS-PAGE loading buffer was mixed, the protein was adsorbed on PVDF membrane by gel electrophoresis, sealed with 5% skim milk, and incubated with primary antibodies BDNF (28205-1-AP, 1: 1000, proteintech), 5-HT (ab85615, 1 *μ*g/mL, Abcam), SHH (20697-1-AP, 1: 1500, proteintech), GLI (66905-1-Ig, 1: 3000, proteintech), PTCH (#2468, 1: 1000, proteintech), SMO (66851-1-Ig, 1: 4000, proteintech), and *β*-actin (66009-1-Ig, 1: 5000, proteintech). Afterward, HRP secondary antibody was incubated. ECL chemiluminescence solution (K-12045-D50, Advansta, USA) was used for chromogenic exposure with *β*-actin as an internal reference to detect expression levels.

### 2.8. Immunofluorescence (If)

The colocalization of BrdU+DCX and BrdU+NeuN in hippocampus was detected by IF. The slices were baked at 60°C for 12 h, dewaxed to water, and the antigen was repaired by heat. The slices were placed in sodium borohydride solution at room temperature for 30 min, and rinsed with water for 5 min. The slices were placed in Sudan black dye solution at room temperature for 5 min, rinsed with water for 3 min, and sealed with 5% BSA for 60 min. The slices were incubated with BrdU (ab8152, 1 : 50, Abcam), DCX (ab18723, 1 : 50, Abcam), and NeuN (26975-1-AP, 1 : 50, PTG) primary antibodies overnight at 4°C. Fluorescence secondary antibody was added and incubated at 37°C for 90 min, and DAPI was stained at 37°C for 10 min. The tablets were sealed and observed under the fluorescence microscope.

### 2.9. 16S rDNA High-Throughput Sequencing

Fecal samples were collected from each group, and fecal DNA was extracted. The extracted total DNA was quantified using Nanodrop (ND-1000, USA), and 1-1.5% agarose gel was used for quality control, such as DNA integrity. Subsequent PCR amplification was performed to obtain variable regions of prokaryotic 16S rDNA to construct high-throughput libraries. Then, the composition and distribution of gut microbiota were detected.

### 2.10. Metabolomics Analysis

Fecal samples were collected from each group, and differential metabolites were analyzed by nontargeted metabolomics based on the ultrahigh-performance liquid chromatography coupled with mass spectrometry (UPLC-MS). UPLC system (Agilent Technologies, China) and Q Exactive Orbitrap (Thermo Fisher Scientific, USA) were used for analysis. Substance identification was carried out using Compound Discover (version 2.0, Thermo, USA) and OSI-SMMS software (version 1.0, Dalian ChemDataSolution Information Technology Co. Ltd) in conjunction with the McCloud database and self-built database. Finally, the normalized data matrix was used for multivariate statistical analysis and screening of differential metabolites.

### 2.11. Statistical Analysis

Graphpad Prism8.0 software was used for statistical analysis. The measurements were expressed as mean ± standard deviation. Normality and homogeneity of variance tests were performed first for conformity to normal distribution and homogeneity of variance. One-way analysis of variance (ANOVA) was used for comparison between multiple groups, followed by Tukey's post hoc test. Pearson correlation coefficient was applied to analyze the correlation between gut microbiota and microbial metabolites. *P* < 0.05 indicated that the difference was statistically significant.

## 3. Results

### 3.1. The Effects of Leonurine on Behavioral Indexes, Inflammation, and Nerve Regeneration in CUMS Rats

**Figure 1:**
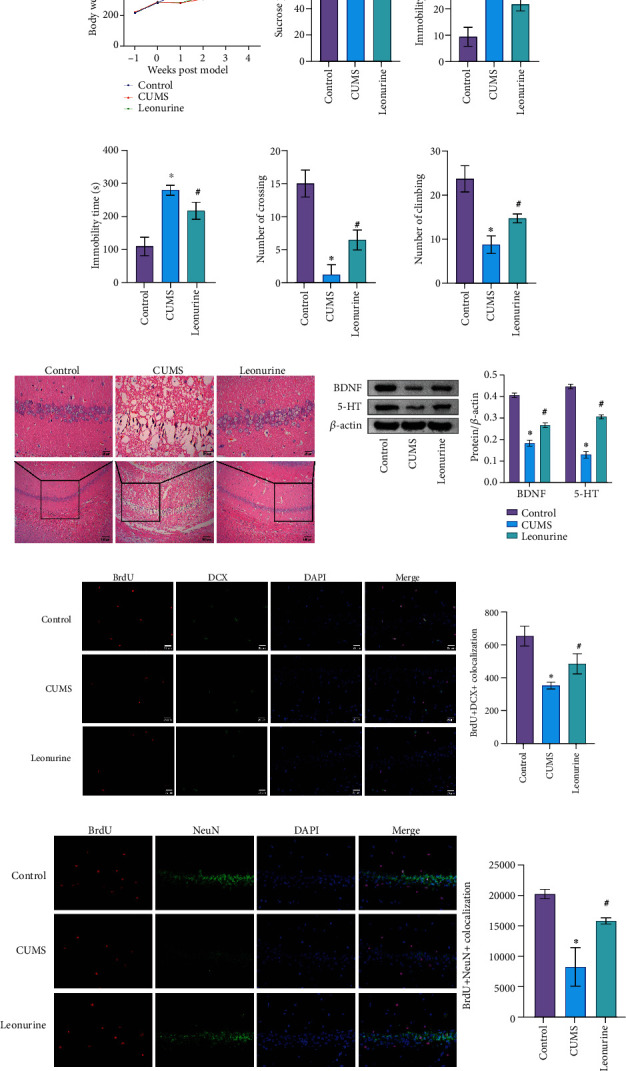
The effects of leonurine on behavioral indexes, inflammation, and nerve regeneration in CUMS rats. (a) Rats body weight assessment. (b) SPT. (c) OFT. (d) FST. (e) HE detection of brain tissue morphology. (f) Western blot detection of BDNF and 5-HT expressions in the hippocampus. (g, h) IF detection of the colocalization of BrdU+DCX and BrdU+NeuN in the hippocampus. ^∗^*P* < 0.05 vs. Control. #*P* < 0.05 vs. CUMS.

To explore the role of leonurine, we first constructed CUMS rats model and conducted a series of tests. As shown in Figure 1(a), compared with the Control group, the body weight of rats was decreased after CUMS modeling, while the body weight was increased after leonurine treatment. Sucrose preference was reduced in the CUMS group compared to the Control group. However, sucrose preference was increased after leonurine treatment (Figure 1(b)). OFT and FST results showed that the immobility time of rats was increased after CUMS modeling, and the numbers of crossing and climbing were decreased compared with the Control group. The immobility time was decreased and the numbers of crossing and climbing were increased after leonurine treatment (Figures 1(c) and 1(d)). HE staining results showed that hippocampal neurons in the Control group had complete structure, regular cell morphology, normal intercellular space, neat arrangement, and clear nucleoli. In the CUMS group, neurons were atrophic and loosely arranged, intercellular space was enlarged, some cells were vacuolated, and the nuclei were pyknotic. The number of hippocampal neurons in the Leonurine group was increased, the intercellular space was narrowed, the arrangement was more orderly, and the histopathological changes were improved (Figure 1(e)). In addition, the expressions of BDNF and 5-HT were decreased after CUMS modeling, and the expressions of BDNF and 5-HT were increased after leonurine treatment (Figure 1(f)). Afterwards, we used IF to detect the colocalization of BrdU+DCX and BrdU+NeuN in the hippocampus. The colocalization of BrdU+DCX and BrdU+NeuN were decreased after CUMS modeling, and the colocalization of BrdU+DCX and BrdU+NeuN were increased after leonurine treatment (Figures 1(g) and 1(h)). These results revealed that leonurine alleviated depressive symptoms and hippocampal nerve regeneration in CUMS rats.

### 3.2. The Effects of Leonurine on SHH/GLI Signaling Pathway

**Figure 2:**
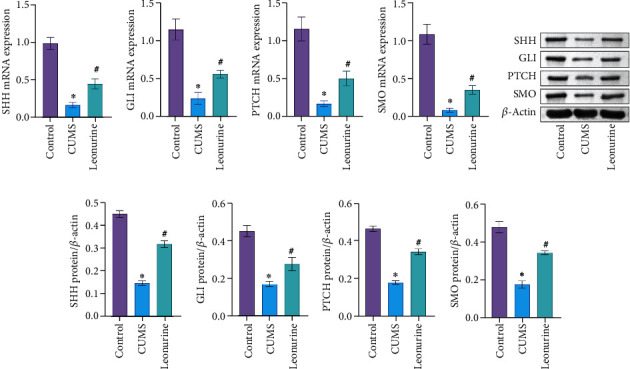
The effects of leonurine on the SHH/GLI signaling pathway. (a–i) SHH, GLI, PTCH, and SMO expressions in the hippocampus were measured by qRT-PCR and Western blot. ^∗^*P* < 0.05 vs. Control. #*P* < 0.05 vs. CUMS.

Previous studies have shown that SHH/GLI signaling pathway may be closely related to neural development in CUMS rats [14, 15]. Therefore, we wanted to further explore the effect of leonurine on the SHH/GLI signaling pathway. We found that the mRNA and protein levels of SHH, GLI, PTCH, and SMO were decreased after CUMS modeling compared to the Control group. After leonurine treatment, the mRNA and protein levels of SHH, GLI, PTCH, and SMO were increased (Figures 2(a)–2(i)). These results suggested that leonurine attenuated depressive symptoms and hippocampal nerve regeneration in CUMS rats via SHH/GLI signaling pathway.

### 3.3. The Effects of Leonurine on Gut Microbiota in CUMS Rats

**Figure 3:**
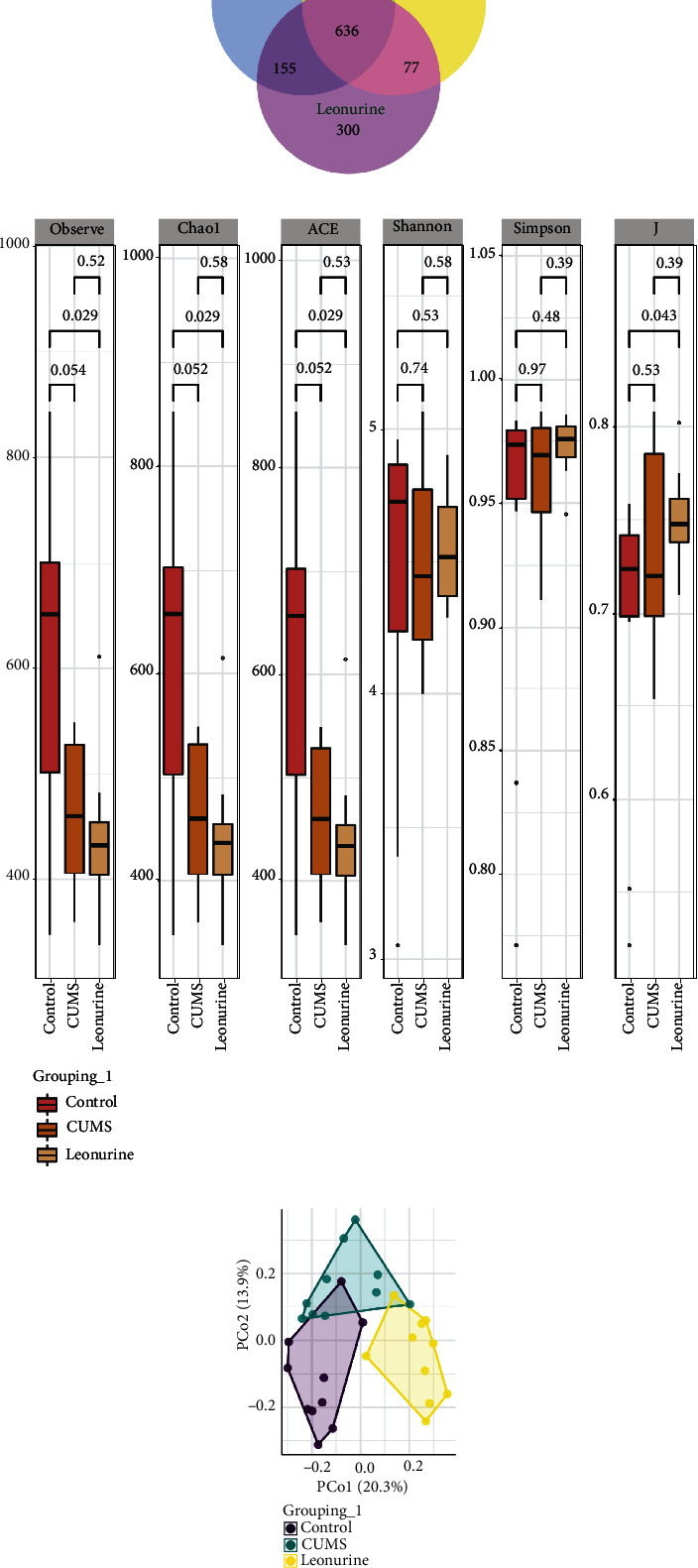
The effects of leonurine on gut microbiota in CUMS rats. (a) Veen diagram: microbiota comparison of Control, CUMS, and Leonurine groups. (b) Alpha diversity analysis. (c) PCoA analysis.

**Figure 4:**
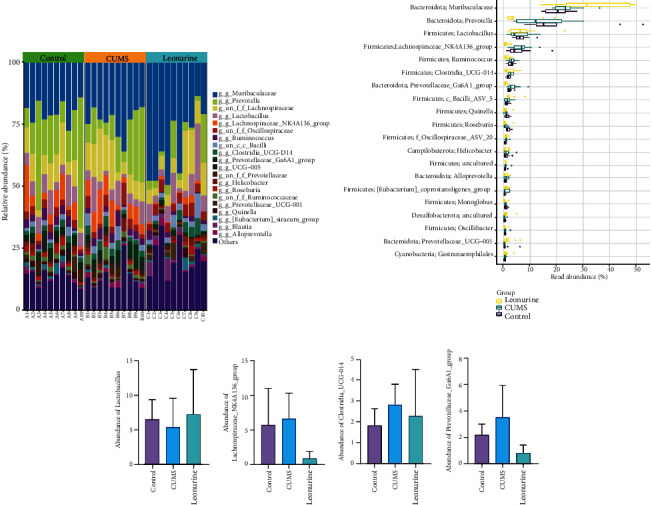
The effects of leonurine on gut microbiota in CUMS rats. (a) Top 20 dominant gut microbiota with relative abundance at the genus level. (b) Relative abundance of microbiota at the genus level. (c–f) Relative abundance comparison of *Lactobacillus*, *Lachnospiraceae_NK4A136_group*, *Clostridia_UCG-014*, and *Prevotellaceae_Ga6A1_group* in the Control, CUMS, and Leonurine groups.

It was reported that gut microbiota could play a role in depression through microbiota-gut-brain axis [16]. Therefore, we wanted to explore the effect of leonurine on the gut microbiota of CUMS rats, and detected the composition and distribution of gut microbiota by 16S rDNA high-throughput sequencing technology. Venn plots showed the common and unique amplicon sequence variants (ASVs) among the groups. Among them, there were 412 unique ASVs in the Control group, 199 unique ASVs in the CUMS group, 300 unique ASVs in the Leonurine group, and 636 common ASVs in all three groups (Figure 3(a)). Alpha diversity was the analysis of species diversity within a single sample. Compared with the Control group, the Observe, Chao1, ACE, and Shannon indexes of the CUMS group were decreased and further decreased in the leonurine group. There was no significant difference between the three groups in Simpson index. However, J index was increased in CUMS group compared to the Control group, and J index was decreased after leonurine treatment (Figure 3(b)). The *β*-diversity was calculated and visualized by PCoA, and as shown in Figure 3(c), there were differences in species abundance among the three groups of samples. The relative abundance histogram of top 20 ASVs at the genus level further showed that the abundance and composition of the microbial community in each group were different. (Figure 4(a)).  Figure 4(b) further showed the relative abundance of the top 20 dominant gut microbiota at the genus level. We found that the abundance of *Lactobacillus* was decreased but the abundances of *Lachnospiraceae_NK4A136_group*, *Clostridia_UCG-014*, and *Prevotellaceae_Ga6A1_group* were increased after CUMS modeling compared to the Control group. Leonurine treatment reversed the abundances of these gut microbiotas (Figures 4(c) and 4(f)). These results indicated that leonurine restored gut microbiota homeostasis in CUMS rats.

### 3.4. The Effects of Leonurine on Microbial Metabolites in CUMS Rats

**Figure 5:**
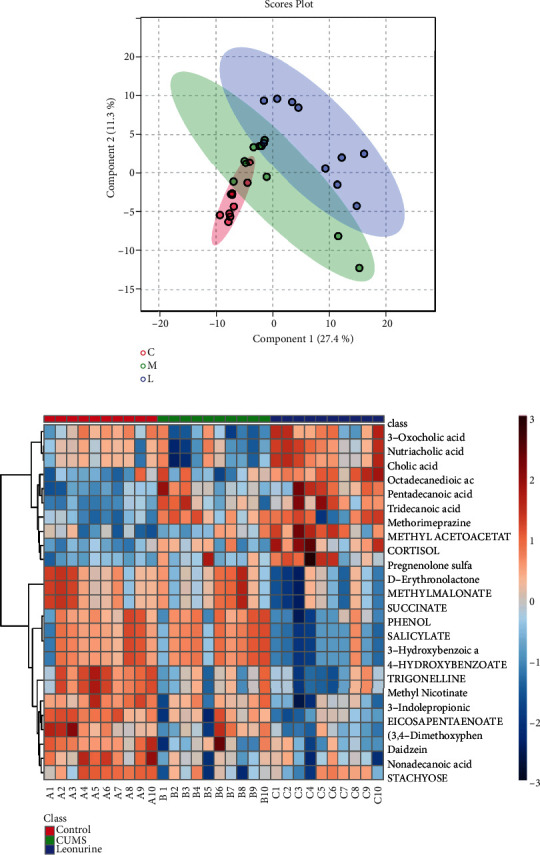
The effects of leonurine on microbial metabolites in CUMS rats. (a) PLS-DA analysis of metabolic profile changes. (b) Heat map showed the metabolic profiles of rats in Control, CUMS, and Leonurine groups.

At the same time, we performed untargeted metabolomics to analyze the differential metabolites. Partial least sums discriminate analysis (PLS-DA) was applied to analyze metabolomic profile changes in the Control, CUMS, and Leonurine groups to eliminate any non-specific effects of this technique and identify relevant biomarkers. Among them, the index of the differential factor variable was 27.4%, which indicated that the three groups of samples were basically separated (Figure 5(a)). Heat map showed that the top 25 potential metabolites in the Control, CUMS, and Leonurine groups. These potential metabolites included 3-Oxocholic acid, nutriacholic acid, and cholic acid (Figure 5(b)). Collectively, leonurine restored microbial metabolite changes in CUMS rats.

### 3.5. Correlation Analysis of Key Gut Microbiota and Microbial Metabolites

**Figure 6:**
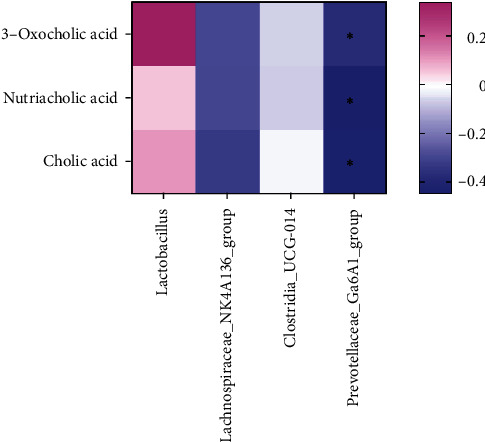
Correlation analysis of key gut microbiota and microbial metabolites. Pearson correlation coefficient analyzed the correlation between gut microbiota and microbial metabolites. ^∗^*P* < 0.05.

In order to investigate whether key gut microbiota and microbial metabolites were correlated, we performed Pearson correlation analysis.  Figure 6 showed that *Prevotellaceae_Ga6A1_group* was negatively correlated with 3-Oxocholic acid (*R* = −0.38, *P* < 0.05), nutriacholic acid (*R* = −0.45, *P* < 0.05), and cholic acid (*R* = −0.44, *P* < 0.05). While *Lactobacillus*, *Lachnospiraceae_NK4A136_group* and *Clostridia_UCG-014* were not correlated with 3-Oxocholic acid, nutriacholic acid, and cholic acid. These results suggested that *Prevotellaceae_Ga6A1_group* might play an important role in CUMS rats.

## 4. Discussion

Depression and related mood disorders pose a huge burden on health, quality of life, and the global economy [24]. Stress-induced failure of the brain's ability to restore plasticity may contribute to the onset and relapse of depression [25]. CUMS is currently the most commonly used, reliable, and valid rodent model of depression [26]. In this study, we performed CUMS rats model to explore the effect and mechanism of leonurine. Our study revealed that leonurine regulated hippocampal nerve regeneration in CUMS rats by activating SHH/GLI signaling pathway and restoring gut microbiota and microbial metabolic homeostasis. This is the first time we report the effect of leonurine on CUMS rats through the SHH/GLI signaling pathway.

Leonurine, the main bioactive component of Herba Leonuri, has shown therapeutic potential in depression [27]. Meng et al. reported that leonurine promotd neurite outgrowth and neurotrophic activity in cultured PC12 cells, and the mechanism may involve GR/SGK1 signaling pathway [9]. Studies have shown that depression-like behavior could be effectively reversed by increasing the expression level of neurotrophic factors in the rat brain [28]. Jia et al. revealed that administration of leonurine for 4 weeks significantly reduced depressive-like behaviors in chronic mildly stressed mice, including increased sucrose preference and decreased immobility time in FST. At the same time, leonurine could effectively restore the level of 5-HT in the hippocampus and prefrontal cortex of chronic mildly stressed mice, and improve the damage of hippocampal neurons [11]. Consistent with their results, our study confirmed that leonurine alleviated depressive symptoms and hippocampal nerve regeneration in CUMS rats.

SHH signaling pathway plays a vital role in the development of many tissues and organs [29]. SHH signaling pathway consists of three main components: PTCH, SMO, and GLI [30]. Abnormal signaling pathways of SHH lead to many neurological diseases, including depression [12]. SHH may be involved in the pathophysiology of depression [31]. However, the mechanism of SHH signaling pathway is complex and still not fully understood. Studies have shown that SHH could regulate adult hippocampal neurogenesis [32]. Mahino et al. reported that SHH expression downregulated in the CUMS induced prenatal stress in the hippocampus [14]. In this study, we treated CUMS rats with leonurine and found that leonurine reversed the reduction of SHH, GLI, PTCH, and SMO levels caused by CUMS. Our study confirmed that the reduction of depressive symptoms and hippocampal nerve regeneration in CUMS rats by leonurine was mediated through SHH/GLI signaling pathway.

TCM may regulate gut microbiota and microbial metabolic homeostasis. Chi et al. demonstrated that oral administration of inulin-type fructooligosaccharides extracted from *Morinda officinalis* alleviated intestinal epithelial damage, restores fecal microbial homeostasis, and relieves depressive symptoms in CUMS rats [33]. Total glycosides isolated from the dried succulent stems of *Cistanche deserticola* alleviated low-grade inflammation of colonic and intestinal barrier disruption, restored disturbed gut microbiota, and then depression-like behavior was reduced [34]. In addition, TCM restored gut microbiota and brain metabolic microbial homeostasis, alleviating depressive behaviors induced by CUMS, and there were strong correlation between brain metabolites and disturbed gut microbiota [35]. In the present study, we also found that leonurine attenuated depressive symptoms and hippocampal nerve regeneration in CUMS rats by regulating gut microbiota and microbial metabolic homeostasis; that was, leonurine restored gut microbiota and microbial metabolite homeostasis in CUMS rats. Moreover, Zhang et al. found the enrichment of *Prevotellaceae-Ga6A1-group* in the gastric microbiota of patients with gastric intraepithelial neoplasia [36]. We also found that *Prevotellaceae_Ga6A1_group* was increased after CUMS modeling and decreased after leonurine treatment. Furthermore, the enrichment of 3-Oxocholic acid in noncachectic patients was correlated with gut microbiota *Lactobacillus gasseri* [37]. Alam et al. identified nutriacholic acid, a novel triterpenoid, in DIBEt for the first time. DIBEt has the potential to defend against oxidative stress stimulation [38]. In addition, cholic acid was increased markedly in plasma of the depression rats [39]. Through correlation analysis, we found that *Prevotellaceae_Ga6A1_group* was associated with 3-Oxocholic acid, nutriacholic acid and cholic acid, which suggested that *Prevotellaceae_Ga6A1_group* might play acrucial role in CUMS rats. Although the current study was conducted in rats, CUMS model we used was widely accepted as an ideal model for studying the antidepressant effects of various potential therapeutic agents. However, the effect of leonurine on SHH/GLI signaling pathway in humans needs to be further investigated.

## 5. Conclusion

In this study, we performed CUMS modeling and explored the role and mechanism of leonurine in CUMS rat model by *in vivo* experiments, 16S rDNA high-throughput sequencing and metabolomics analysis. Our study revealed for the first time that leonurine modulated hippocampal nerve regeneration in CUMS rats via SHH/GLI signaling pathway and restoring gut microbiota and microbial metabolic homeostasis. This study provides a reference for exploring the mechanism of action of leonurine in depression.

## Data Availability

The datasets used and analysed during the current study are available from the corresponding author on reasonable request.
